# Recovery of metallic iron from the loaded organic phase after solvent extraction by precipitation–stripping with hydrogen gas

**DOI:** 10.1039/d6ra00829a

**Published:** 2026-03-04

**Authors:** Clément Laskar, Koen Binnemans

**Affiliations:** a Department of Chemistry, KU Leuven Celestijnenlaan 200F, P.O. Box 2404 3001 Leuven Belgium; b University of Toulouse, CNRS, Toulouse INP, LGC Toulouse France clement.laskar@toulouse-inp.fr

## Abstract

Removal of iron from pregnant leach solutions (PLS) is a critical yet challenging step in hydrometallurgical processing prior to the recovery of valuable metals. Conventional iron removal by precipitation of ferric compounds such as goethite or jarosite generates large amounts of solid waste. Solvent extraction (SX) offers an alternative route, enabling subsequent recovery of iron as a marketable product. In this study, an innovative SX-based iron removal process was investigated. Ferric iron was first extracted from aqueous solution into an organic phase containing the carboxylic acid extractant Versatic Acid 10 (VA10). The loaded organic phase (5–16 g Fe per L) was then subjected to direct reduction using hydrogen gas to precipitate iron. The precipitation–stripping rate was enhanced by adding a base (Mg(OH)_2_ or NH_3_) at 200 °C and H_2_ pressures up to 10 bar, with reaction times of 2 to 16 hours. The effect of different seeding materials (Ni, C and Fe) on iron precipitation was examined. Formation of metallic iron particles was observed only with Ni seeds, a high H_2_/Fe molar ratio (≥12), and the addition of either Mg(OH)_2_ or NH_3_. Under comparable conditions, precipitation yields with Ni seeds were up to 16 times higher than with carbon seeds. VA10 degradation was lower with Mg(OH)_2_ than with NH_3_. At lower H_2_/Fe molar ratios, regardless of seed type or base addition, only iron oxides (magnetite and hematite) were formed, demonstrating the need for an excess of hydrogen gas well above stoichiometric requirements.

## Abstract 2

Stripping of iron(iii) with hydrogen gas from a loaded solvent after extraction with Versatic Acid 10 leads to formation of either metallic iron or magnetite, depending on the experimental conditions.

## Graphical abstract

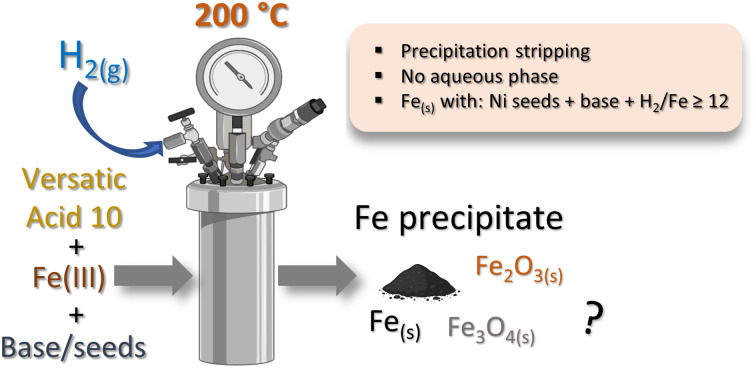

## 1. Introduction

Hydrometallurgy involves the extraction of metals from ores, concentrates, industrial intermediates and end-of-life products, along with the refining of these metals through aqueous processes. The distinguishing feature of hydrometallurgy is the use of aqueous solutions, setting it apart from pyrometallurgy, which relies on high-temperature processes. While hydrometallurgy allows for the production of very pure metals, iron is a problematic impurity.^1–3^ This chemical element is present in high concentrations in ores and concentrates of copper, zinc, and nickel, or lead smelter residues.^4^ Sulfidic zinc concentrates contain 10 to 15 wt% of iron, chalcopyrite (CuFeS_2_) concentrates contain one iron atom for each copper atom present, and iron is the main component of limonitic nickel laterite ores. Iron is co-dissolved with the metals of interest when acids are used for leaching. The dissolved iron is typically removed from the *pregnant leach solution* (PLS) by neutralisation of the solution to precipitate iron(iii) hydroxides (ferrihydrite or goethite),^5^ or basic iron(iii) sulfates (jarosite).^6,7^ These precipitates are bulky, and solid/liquid separation is difficult. The best way to resolve this problem is to convert the iron into hematite (Fe_2_O_3_) with a purity acceptable for steelmaking or pigments, or into metallic iron.^8–11^ Several studies have also focused on the direct reduction of Fe_2_O_3_ to metallic iron in alkaline or acidic electrolytes, but much improvement is still needed in terms of energy efficiency by impurity control.^12–14^

A pure iron product can be obtained with high efficiency only from purified solutions.^15^ The *Best Available Technology* (BAT) for iron purification is *solvent extraction* (SX), also known as liquid–liquid extraction.^16,17^ SX is based on the selective distribution of metal ions between an aqueous phase and an immiscible organic phase. The organic phase comprises the extractant, which is the active compound for transferring metal ions to the organic phase, and the diluent, which dissolves the extractant and the extracted metal complexes, and controls the viscosity and density of the organic phase. A phase modifier can be added to avoid third-phase formation and to facilitate phase separation. The purification of iron through SX, followed by recovery of iron as pure hematite or metallic iron, is regarded as the most promising solution to the iron problem.^18–20^ Most studies on SX of iron are fundamental ones and focus on extraction and stripping, neglecting further downstream processing to a marketable product such as hematite or metallic iron.^21^ The kinetics of Fe(iii) extraction are slow and Fe(iii) is difficult to strip from the loaded organic phase of acidic extractants, compared to Fe(ii).^22^ A 6 M HCl solution or a reducing agent is required for stripping from a loaded organic phase with bis(2-ethylhexyl)phosphoric acid (D2EHPA) extractant.^23,24^ Another, but industrially still unproven, method for iron removal from the loaded organic phase is hydrolytic stripping. In this process, the iron-loaded solvent is reacted with water in batch reactors (autoclaves) at temperatures between 150 and 200 °C to precipitate hematite directly from the organic phase with simultaneous regeneration of the acid extractant in its protonated form.^25,26^ The primary obstacles that hamper further development of this process are the slow extraction kinetics, the high energy costs associated with the thermal hydrolysis process, and the particle properties, *i.e.*, poor settling of the precipitate and poor quality of the hematite product. The observed particle size depends on the process temperature, increasing from 20 µm at 170 °C to 40 µm at 215 °C; but unfortunately, solvent decomposition is observed at higher temperatures.

**Fig. 1:**
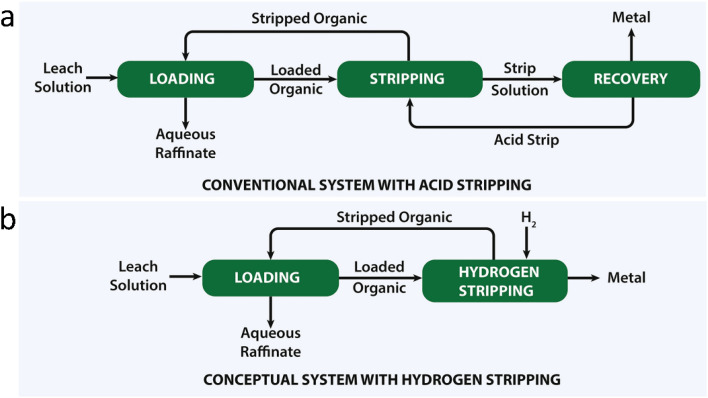
Conventional solvent-extraction flowsheet in which the loaded organic phase is stripped and the metals are subsequently recovered from the aqueous strip solution (a), compared to the conceptual flowsheet studied in this work where metal recovery occurs *via* pressure-hydrogen stripping directly from the loaded organic phase (b). Adapted from ref. 28.

Another industrially still unproven method inspired by hydrolytic stripping is hydrogen stripping, where iron is reduced to the metallic state by hydrogen gas.^27,28^ This method has the advantages of obtaining pure metal and avoiding the oxidation of the extractant in a two-step process with no need for an intermediate stripping solution (Fig. 1). The formation of Cu, Co and Ni metal powder in the organic phase after SX has been observed,^29,30^ but for these metals the production of metal powders by reduction with hydrogen gas is also feasible in aqueous solutions.^31^ The most challenging reduction with is that to iron metal,^29^ because the reduction of iron species in aqueous solution beyond Fe(ii) is impossible from a thermodynamic point of view in aqueous solutions.^17^ However, the situation is different in organic solutions where the Fe(iii) is not hydrated by water molecules. The proof-of-concept for this process was demonstrated more than 50 years ago by Burkin, but it has never been investigated in detail nor confirmed by independent research.^32^ Burkin reported iron precipitation under high temperature and pressure using a carboxylic acid (Versatic Acid 911, analogous to Versatic Acid 10 but containing C9–C11 acids, rather than only C10 isomers) in a hydrocarbon diluent, with temperatures up to 300 °C, hydrogen pressures up to 68 bar, ammonia as a base, and carbon seeds.^32–34^ Despite promising preliminary observations, no definitive evidence of metallic iron formation was provided. The precipitate, initially assumed to be iron metal due to its pyrophoric behaviour, was later identified by XRD as magnetite (Fe_3_O_4_), with the authors suggesting that any metallic iron had been oxidised upon exposure to air.^33^ More recently, precipitation of iron metal from iron(iii) acetylacetonate by hydrogen reduction was also performed but in acetylacetone solvent,^35^ with obtention of iron metal nanoparticles.^36^ Versatic Acid 10 (VA10), which is a commercial mixture of branched carboxylic acids with 10 carbon atoms, appears to be the best extractant for this application, because of its higher thermal stability than D2EHPA.^17^ The use of the extractant bis(2,4,4-trimethylpentyl)phosphinic acid (Cyanex 272) is also more complicated due to its higher viscosity so that solid/liquid separation is more difficult, with more impurities that can react, even if this is also a very stable commercial acidic extractants at high temperature (∼200 °C).^37^ Selecting VA10 also avoids the leaching of iron oxides that could potentially form, because Fe_2_O_3_ and Fe_3_O_4_ are insoluble in it.^38^

The present study aims to build upon Burkin's pioneering work by assessing whether precipitation–stripping of iron using hydrogen gas is a viable route to recover metallic iron, and to identify the mildest experimental conditions. In the present work, the effects of base addition—either ammonia (NH_3_) or magnesium hydroxide (Mg(OH)_2_)—and seeding on precipitation yields and kinetics are systematically investigated to improve the efficiency of metallic iron recovery under controlled conditions.

## 2. Experimental

### 2.1. Materials

Solids: iron(iii) sulfate pentahydrate (97%, Thermo Scientific); iron metal-type I (325 mesh, 97%, Aldrich); iron metal-type II (20 mesh, 99%, ABCR); Ni metal (<50 µm, 99.7%, Sigma-Aldrich); magnesium hydroxide (95–100.5%, Chem-Lab); activated charcoal (20–40 mesh, DARCO, Aldrich). Liquids: Versatic Acid 10 (Hexion); D2EHPA (Acros Organics); Cyanex 272 (Syensqo); Shellsol G80 (Shell Chemicals Europe); 1-butanol (>99.4%, Merck); 1-decanol (99%, Merck); ICP standards of Fe, Ni, Mg (1000 mg L^−1^, in 2–5 wt% HNO_3_, Chem-Lab). Gas: ammonia (99.9%, AirLiquide). All chemicals were used as received, without any further purification.

### 2.2. Instrumentation

The high-pressure, high-temperature (HP-HT) experiments were carried out in three parallel, 78 mL cylindrical Hastelloy reactors (HSTC, Ni–Mo–Cr alloy, Parr Industry) in a Parr Industry Multiple High-Pressure Leaching Reactor System (Series 5000) with internal magnetic stirring (0–1200 rpm). The magnetic stirrer had a slightly smaller diameter than the reactor to ensure stirring and prevent solid accumulation in dead volumes. The sealing of the reactor was ensured with the help of PTFE gaskets (5050/51 grade, Parr Industry). Temperature was measured directly with a temperature probe in the liquid to attain the desired final temperature in the reactor. Pressure was measured in the reactor with a pressure gauge.

The concentrations of Fe, Ni and Mg in organic solutions were determined by inductively coupled plasma optical emission spectroscopy (ICP-OES). The Avio 500 spectrometer (PerkinElmer, USA) was equipped with a GemCone low-flow nebuliser, a baffled cyclonic spray chamber, an organics-compatible standard injector, and a PerkinElmer 3-slot Hybrid XLT torch. All samples were filtered using a 0.5 mL syringe with a 0.45 µm syringe filter prior to dilution, to prevent the analysis of residual solids and the contamination of analytical instruments with any residual suspensions within the liquid. All samples (including quality controls and calibration standards) were diluted with 1-butanol to reach the desired concentration, then measured in triplicate. The concentrations analysed for all samples were within the range of the calibration standards, for which four standards were used (0.50, 1.0, 5.0 and 10 ppm for the Fe, Ni and Mg elements). The solids formed in the organic phase were characterised to determine their chemical composition, purity, morphology and particle size distribution. X-ray diffraction (XRD) analysis was performed using a D2 Phaser X-ray diffraction spectrometer with Cu-Kα X-ray radiation (30 kV; 10 mA) at an angle of 2*θ* between 20° and 100°. A direct analysis using scanning electron microscopy coupled with energy-dispersive X-ray spectroscopy (SEM-EDX) was performed to investigate the nature of the particles' iron content. SEM-EDX was performed using a Tescan MIRA 4 FEG-SEM with an Oxford 30 mm^2^ EDX detector. EDX analyses were performed using Kα1 energy to quantify the elements Fe, O and Ni, which have no overlapping peaks in the conditions used, operating at 20 keV and a working distance of 15 mm. High-performance liquid chromatography (HPLC) with a Shimadzu LCMS-2020 system with a DUIS-2020 dual ion source in ESI/APCI± mode was used to investigate the degradation of the organic solution, particularly that of the acidic extractant VA10. The column was an InfinityLab Poroshell 120 EC-C18, 2.7 µm, 2.1 × 100 mm. The samples were diluted 400-fold with MeOH/DCM (9 : 1) and 1 µL was injected for analysis. The eluent composition varied from 100 vol% methanol in water (containing 0.1% formic acid) to 100 vol% methanol (containing 0.1% formic acid), over a period of 12 minutes, followed by 6 minutes at 100 vol% methanol, providing satisfactory performance at a flow rate of 0.350 mL min^−1^ to ensure stable ionisation and efficient desolvation in the source. The wavelength range of the photodiode array (PDA) detector was from 190 to 800 nm.

### 2.3. Methodology

The organic solutions (Fe: 3–16 g L^−1^; VA10: 10–30 vol% in G80), which were added to the reactor, were prepared by loading Fe(iii) from aqueous solutions. This range of iron concentrations was chosen to ensure that a sufficient quantity of iron precipitate could be recovered for experimentation, while also ensuring that the level of extractant remained low enough to prevent the formation of a third phase. To transfer the Fe(iii) into the organic solution, the organic phase was first loaded with Mg(ii) by an acid–base reaction between Mg(OH)_2_ and VA10, according to the reaction:1
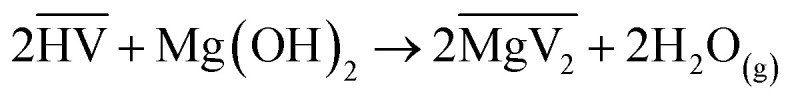
here VA represents VA10 and V the corresponding versatate anion. The Mg(OH)_2_ was dissolved directly in 300 mL of VA10 + G80 at a temperature of 70 °C for three hours, with stirring at 800 rpm (3.5 g of Mg(OH)_2_ in 300 mL for 30 vol% VA10 and 1.2 g of Mg(OH)_2_ in 300 mL for 10 vol% VA10 + G80). The entire solution was then filtered using a 1.6 µm glass fibre filter (4.7 cm diameter) to remove any excess Mg(OH)_2_ in the liquid (<0.05 g). The Mg-loaded organic phase was then mixed with the Fe(iii) aqueous solution (10 g L^−1^ Fe for 30 vol% VA10 and 3.3 g L^−1^ for 10 vol% VA10 + G80) at an organic-to-aqueous volume ratio of 1 : 2, with stirring at 1400 rpm and a temperature of 25 °C. As Fe(iii) is preferentially loaded in the VA10 solution over Mg(ii), the exchange reaction is:^39^2
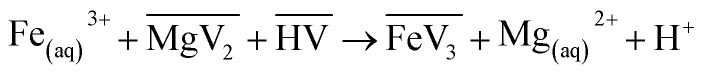


A dark brown solution was obtained, similar to the Fe-loaded organic solution described by Burkin,^33^ compared to the colourless solution containing only VA10 or the Fe(ii) ion. The initial Fe concentrations varied depending on how long the Mg and Fe solutions were left to mix for (from 2 to 30 hours). The mixture was then left to settle and the organic phase was recovered and filtered using a 1.6 µm glass fibre filter to remove any residual solid in suspension. In experiment V2-5, 5 vol% of 1-decanol was added to prevent the formation of a third phase that occurred upon the addition of NH_3_.^17^ In experiment V5-3, a larger amount of extractant (30 vol%) was used to minimise the influence of NH_3_ on the formation of a third phase. This test was then performed without 1-decanol to investigate extractant degradation only, and no third phase formation was observed. The HP-HT reactors were loaded with between 15 and 40 mL of organic solution. Depending on the experiment, solid seeds and a solid (Mg(OH)_2_) or gaseous (NH_3_) base were added. Seeds were added to aid precipitation, using Ni, Fe or C (activated charcoal) seeds. In Burkin's work with Versatic Acid 911, graphite was used as a seed to precipitate iron.^33^ Nickel seeds were previously used to reduce Fe(iii) to Fe(ii) in an organic D2EHPA solution.^17^ Two types of iron seeds were tested in our experiments to investigate possible differences. Before loading any gas (NH_3_ and H_2_) in the reactor, 10 consecutive flushes at 1.4 bar of nitrogen gas were performed to remove all air, especially oxygen gas, from the sealed reactor. NH_3_ gas was added with one flush of 4 bar in experiments V2-4 and V2-5, and five flushes in experiment V5-3, to attain saturation in the fluid with quick dissolution of the gas in the reaction within a minute. The viscosity of the liquid increases with the addition of NH_3_, which can be observed if the reactor is opened immediately after NH_3_ loading, because some complexes are formed with VA10.^40^ The elevated viscosity may complicate post-reaction filtration compared with experiments performed using Mg(OH)_2_. However, the use of ammonia gas facilitates rapid and homogeneous dissolution of the base in the liquid phase. Hydrogen was loaded at a pressure of 10 ± 0.1 bar for all experiments. This high hydrogen pressure ensures a stoichiometric excess of H_2_ over Fe (1.4–23). The temperature was set at 200 ± 1 °C for all experiments, as a compromise between the quality of sealing due to the thermal resistance limit of the PTFE gasket, the degradation of the extractant,^34^ and favourable kinetic conditions.^17^ After 2–16 hours at 200 °C, depending on the experiment, the reactor was cooled for 1 hour. The hydrogen gas was removed by flushing three times with nitrogen gas. After opening of the reactor, the entire suspension was filtered through a 1.6 µm glass fibre filter to allow for the rapid filtration of organic liquids and the recovery of precipitated solids. Due to their ferromagnetic nature, we took care to recover all the precipitate from the walls and magnetic stirrer, as they are automatically attracted to them. The solid was rinsed with ethanol (2 × 5 mL). The solid was dried under vacuum at 50 °C for two hours, except for experiments V5-1, V5-2 and V5-3, where the solid was recovered in a glovebox under a nitrogen atmosphere and left to dry for 24 hours in the glovebox to prevent the recovered iron solid from oxidising following the opening of the reactor. The dried solid was then stored in a pill under a nitrogen atmosphere until it was characterised by XRD and SEM-EDX. As most of the precipitate was stuck in the filter fibres, XRD analysis was performed directly on the filter. XRD analyses were performed within one minute of opening the pill to avoid oxidising the solid upon contact with air. The composition of the residual organic solution in terms of Fe, Mg and Ni (in the case of the use of Ni seeds) was analysed by ICP-OES with 1000-fold dilution. The iron precipitation yield was calculated as follows:3
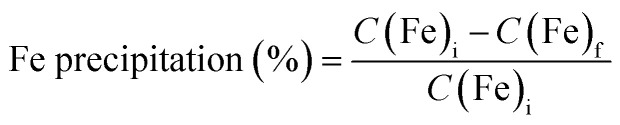
where *C*(Fe)_i_ is the initial concentration of iron in the organic solution loaded with Fe(iii) and *C*(Fe)_f_ is the final concentration of iron in the organic solution after the reaction. For all experiments, the pressures were lower than with water (*e.g.*, for hydrolytic stripping) due to the low vapour pressure of the organic solvent, as we only had organic solution. Pressures were always measured below 16 bar at 200 °C, while an initial pressure of 10 bar of H_2_ was added at 25 °C before heating. The absence of leaks was verified by always checking the pressure all along and at the end of the experiments. As Burkin's work suggested that the limiting experimental parameter was the transport of hydrogen in the solution,^33^ a higher stirring rate (1000 rpm *vs.* 600 rpm) was also applied in the final experiments (V4 and V5 series) because the initial loading volumes of the organic solutions were higher.

## 3. Results and discussion

All the experimental details and main results are summarised in Table S1 and the iron precipitation results with the main parameters are shown in Table 1. Firstly, we compared the three acidic extractants (VA10, D2EHPA and Cyanex 272) with and without Mg(OH)_2_ as a base (Table S1). Experimentally we observed that the fluid was too viscous with Cyanex 272 (C1-1 and C1-2), resulting in the formation of a third phase. Furthermore, as experiments using Mg(OH)_2_ as a base gave significantly higher precipitation yields with VA10 at 200 °C (38.1 ± 0.3% Fe, V1-2) than with D2EHPA (12.3 ± 0.1% Fe, D1-2) at 150 °C (to avoid its thermal degradation), with molar ratios Base/Fe > 1 and H_2_/Fe ≥ 12 in both cases, we focused for the following experiments with only VA10 as the extractant. During all subsequent experiments (V2, V3, V4 and V5 series), three parameters were varied in order to investigate their influence on the yield of iron precipitation and the nature of the precipitate: the iron concentration (ranging from 5 to 16 g L^−1^), the presence of a base (Mg(OH)_2_, NH_3_ or no base), the presence of seeds (Ni, C, Fe or no seeds). Temperature was set, and experiment's duration has been changed to try to increase the precipitation yields of iron. The changes of volume of the organic phase loaded and of the iron concentration implied variations in the H_2_/Fe molar ratio. Two key results were considered: the iron precipitation yields and the nature of the iron precipitated particles (metal and/or oxides).

**Table 1:** Precipitation yields of Fe and nature of precipitated particles after the experiments with VA10 as an extractant. See Table S1 for more details

#	*C*(Fe)_i_ (g L^−1^)	Fe precipitation (%)	Seeds type	Base type	Base/Fe excess^a^	H_2_/Fe excess^a^	Time (h)	Precipitated particles
V1-1	2.72 ± 0.01	No	Fe-type I	—	—	23	2	N.A.
V1-2	2.67 ± 0.01	38.1 ± 0.3	Fe-type I	Mg(OH)_2_	1.3	23	2	N.A.
V2-1	5.04 ± 0.01	4.1 ± 0.1	Ni	—	—	12	2	Fe metal
V2-2	5.04 ± 0.01	12.6 ± 0.3	Ni	Mg(OH)_2_	1.3	12	2	Fe metal
V2-3	5.04 ± 0.01	62 ± 1	Ni	Mg(OH)_2_	1.3	6.1	2	Fe_3_O_4_
V2-4	5.04 ± 0.01	—b	Ni	NH_3_	2.8	12	2	Fe metal
V2-5^c^	5.02 ± 0.01	9.2 ± 0.1	Ni	NH_3_	2.8	12	2	Fe metal
V3-1	5.18 ± 0.03	No	C	—	—	7.3	2	N.A.
V3-2	5.18 ± 0.03	3.8 ± 0.2	C	Mg(OH)_2_	1.3	7.3	2	Fe_3_O_4_
V4-1	12.18 ± 0.02	28.8 ± 0.3	Fe-type I	Mg(OH)_2_	0.8	1.9	16	Fe_3_O_4_
V4-2	12.18 ± 0.02	28.1 ± 0.4	Fe-type II	Mg(OH)_2_	0.8	1.9	16	Fe_3_O_4_
V4-3	12.18 ± 0.02	30.4 ± 0.4	—	Mg(OH)_2_	0.8	1.9	16	Fe_3_O_4_
V5-1^d^	16.06 ± 0.02	No	—	—	—	1.4	16	N.A.
V5-2^d^	16.06 ± 0.02	21.9 ± 0.1	—	Mg(OH)_2_	0.6	1.4	16	Fe_3_O_4_
V5-3^d^	16.06 ± 0.02	43.4 ± 0.4	—	NH_3_	0.7	1.4	16	Fe_2_O_3_ + Fe_3_O_4_

a Excess molar ratios are calculated according to the stoichiometry of reactions (4) and (5).

b No titration was possible due to the formation of a third phase. In this case, XRD analysis was not feasible.

c Decanol (5% vol.) was added as a phase modifier to avoid the formation of a third phase.

d The solid was recovered in a glovebox to avoid its oxidation.

### 3.1. Effect of the seeds and experimental planning

The two main ideas behind using seeds in our experiment were, first to conduct experiments with carbon (V3 series) and iron seeds (V1 and V4 series) in an attempt to precipitate iron metal while avoiding contamination with other metals; and secondly, to conduct experiments with nickel seeds (V2 series) because they are highly reactive.^17^ Iron seeds are stable in organic acid because experiments with iron seeds (V1-1, V1-2, V4-1 and V4-2) showed no over-dissolution of iron in the fluid compared to the experiment with no seeds (V4-3). The same applies to Ni seeds, as no Ni concentration was measured by ICP-OES in the liquid during experiments with nickel seeds (V2 series).

The main challenge was the difficulty to recover and analyse a solid that had precipitated if the initial volume load and Fe concentration were too low (experiments V1-1 and V1-2). For these experiments, no XRD and SEM-EDX analysis were performed. This limit makes it difficult to achieve high H_2_/Fe excess molar ratios. This meant that we had to ensure that a sufficient amount of solid precipitated so that the solid could be characterized properly. We needed sufficient amount of iron precipitated particles to produce enough powder for XRD measurements (∼0.1 g). Furthermore, it was not possible to investigate the same conditions with the different seeds. With nickel seeds (V2 series), the precipitated particles were easier to recover due to the particles behaviour and the magnetic properties of nickel seeds which tended to form agglomerates, and the precipitation yields of iron were high. With carbon seeds (V3 series), the precipitate amount was too low because of low precipitation yields.

As insufficient powder was recovered in experiments involving precipitation with low initial volumes (experimental volume < 20 mL, *C*(Fe)_i_ ≤ 5.2 g L^−1^), further experiments were conducted with higher iron concentrations (*C*(Fe)_i_ ≥ 12 g L^−1^), larger volumes (40 mL) and longer time (16 h) to examine the nature of the precipitated iron particles (V4 and V5 series), without the addition of nickel seeds which adds pollution from a metal other than iron.

### 3.2. Effect of adding a base on the precipitation of iron

Two different bases were tested: NH_3_(g) and Mg(OH)_2_. Adding these bases helped to shift the equilibrium of this very slow kinetic reaction to the right in the case of precipitation of iron particles.^29^ The relevant reactions are:4
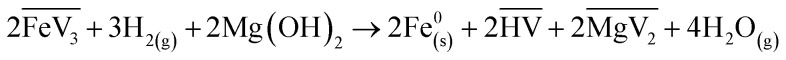
5
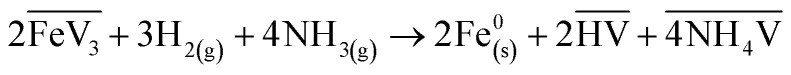
where V represents the anion of the extractant Versatic Acid 10. In the presence of Mg(OH)_2_, the pressure was slightly higher (by 0.1 to 1 bar) at the end of the experiments, which is potentially due to the formation of gaseous H_2_O during the decomposition reaction of Mg(OH)_2_. In all experiments where Mg(OH)_2_ was added, the Mg concentrations showed total dissolution of the pre-loaded Mg(OH)_2_ solid. In Burkin's experiments, ammonia gas was added to push the reaction to completion, with no Fe precipitation observed without the addition of this base.^32,33^ An advantage of using Mg(OH)_2_ compared to NH_3_ is that the final Mg-loaded/Fe-depleted organic solution can be reloaded with Fe from an aqueous Fe(iii)-rich PLS. Magnesium oxide or hydroxide (in alkaline solution) can then be recovered from the Mg-rich aqueous solution by evaporating the water, which is coherent with the aim of developing the most circular process.^28^ NH_3_ is more complicated to recover due to the need of solvent regeneration at high temperatures (>100 °C).^41^

Firstly, it is clear that adding a base increases the yield of iron precipitation compared to similar experiments without the addition of a base (see Table S1). With the addition of nickel seeds, an increase in the iron precipitation yield was measured by a factor of 3.1 with Mg(OH)_2_ (experiment V2-2) and by a factor of 2.2 with NH_3_ (experiment V2-5) compared to the experiment without a base (experiment V2-1). With the addition of carbon seeds (experiments V3-1 and V3-2), the precipitation only occurred in the presence of a base (Mg(OH)_2_). The same observation occurred with no seeds and no base where no iron precipitation occurred (experiment V5-1), while a precipitation was observed with a base (experiments V5-2 and V5-3). It should be noted that it is difficult to quantitatively compare the efficiency of Mg(OH)_2_ and NH_3_ in helping to precipitate iron directly from our results, because the base/Fe excess according to the stoichiometry of reactions (4) and (5) are not the same, and the details of the mechanisms of these reactions are not known. Furthermore, the fact that iron oxides are obtained in both experiments with NH_3_ (experiment V5-3) and Mg(OH)_2_ (experiment V5-2) for similar conditions shows that oxidation is not due to the presence of oxygen from Mg(OH)_2_. Considering the results obtained using nickel seeds and Mg(OH)_2_ as a base with similar conditions, but with a difference in the H_2_/Fe molar ratio (experiments V2-2 and V2-3), it appears that iron is more easily precipitated in the form of oxides than in its metallic state. The yield of iron precipitation is 4.9 times higher at a H_2_/Fe molar ratio 2 times lower, but iron oxide (Fe_3_O_4_) is obtained. It should be noted that, since the final precipitation yields were the same with and without iron seeds (experiments V4-1, V4-2 and V4-3), no experiments were conducted with iron seeds added without a base and tests with no seeds were favoured in the last experimental series (V5).

### 3.3. Characterisation of the iron precipitate

**Fig. 2:**
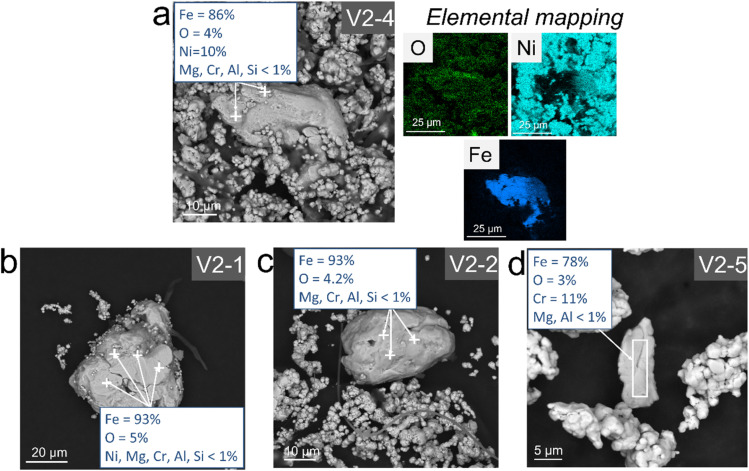
SEM microphotographs taken in back-scattering mode (BSE mode), and elemental composition analysis performed using EDX. For clarity, an elemental map has been created for the V2-4 sample (a), as the iron metal chunk is surrounded by nickel metal seed particles. Fe metal precipitates only occur in the presence of Ni metal seeds. The back-scattering electron mode was used to more clearly observe the iron metal particles. The chromium impurity in the V2-5 precipitate (d) may come from the reactor alloy. See Tables 1 and S1 for experimental details.

**Fig. 3:**
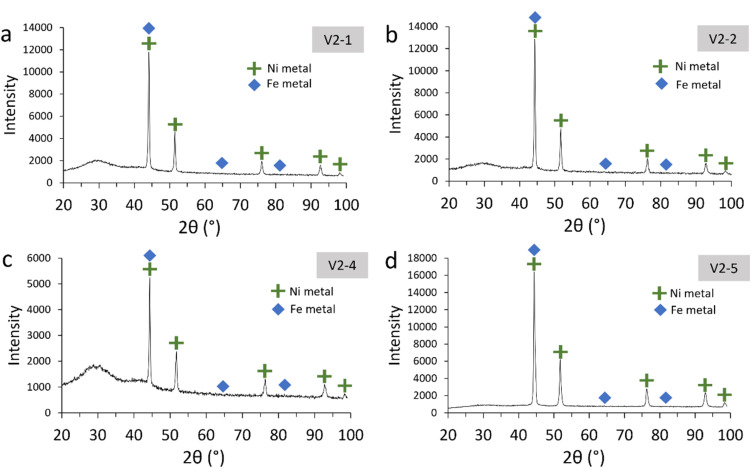
XRD diffractograms of the precipitates after the experiments, in which Fe metal was observed using SEM-EDX. The main peaks of the Ni metal from the seeds (44.481°) and the Fe metal precipitate particles (ferrite, α-Fe, 44.354°) overlap, which makes it impossible to distinguish between them. However, no peaks from Fe oxides were visible. The structures reported are iron metal (ferrite, α-Fe) (ICSD 064795)^42^ and nickel metal (ICSD 76667, at 500 K closed to our experimental conditions).^43^ See Tables 1 and S1 for experimental details.

Metallic iron particles obtained directly from a loaded organic phase have been characterised for the first-time using SEM-EDX analysis when nickel seeds and a large H_2_/Fe molar excess (≥12) are used (see Fig. 2, experiments V2-1, V2-2, V2-4 and V2-5). Typically, they have a size between 10 and 20 µm and high iron content (≥78% Fe). In the context of iron metal formation, XRD could not produce evidence for the formation of metallic iron (Fig. 3), because the main iron metal peak (ferrite, α-Fe, stable allotropic form in our conditions) nearly coincides with the main peak of the nickel seeds from our reference material peak data:^42,43^ α-Fe peak (44.354°, 100% relative intensity) compared to Ni peak (44.481°, 100% relative intensity). Consequently, as the amount of precipitated iron particles is significantly lower than that of nickel seeds (molar ratio Fe/Ni ≤ 0.2), and nickel exhibits slightly stronger X-ray scattering due to its higher number of electrons, the main iron peak is likely indistinguishable within the base of the intense main nickel peak. Furthermore, the second most intense α-Fe peak (81.657°, 17.6% relative intensity) is not observed in the diffractograms, further supporting the conclusion that the quantity of precipitated iron is too small to exceed the detection limit. However, it must be noted that no iron oxide peaks were also observed by XRD (Fig. 3), even if it is difficult to ascertain whether this was due to a lack of iron particles or because such iron oxide phases were not precipitating. Magnetite was obtained (Fig. S3a) with a lower H_2_/Fe molar ratio (6.1) and a greater initial amount of iron-rich organic solution in the reactor (V2-3).

The experiment V3-2 involving carbon seeds resulted in the formation of iron oxide visible by SEM-EDX (Fig. S1b). In this case, it would have been possible to detect the presence of iron by XRD, because only a small peak of carbon graphite (44.833°, 10% relative intensity) is at a close angle value than α-Fe main peak (44.354°). However, the amount of iron that precipitated was too low, and only graphite main peak (26.347°) could be observed (Fig. S3b).

The results of both the SEM-EDX and XRD analyses are coherent when iron oxides (magnetite + hematite) are observed (Fig. S1–S3), with Fe seeds, as well as those without seeds. EDX analysis gives a higher Fe/O ratio than magnetite (Fe and O at% for magnetite are 43% and 57%, respectively). This demonstrates that some iron metal may co-precipitate in small proportions at the grain surface. However, they could not be detected by XRD. Fig. S1 shows that iron oxide precipitates are similar with or without iron seeds, with no observation of iron metal peaks in XRD (Fig. S3). Furthermore, the precipitation yields and the nature of the precipitates obtained (size and shape, Fig. S2) with (V4-1 and V4-2) and without (V4-3) iron seeds are similar, demonstrating that the addition of iron seeds has no effect. The SEM results in Fig. S1 demonstrate that magnetite pellets of a similar shape to those obtained in previous studies were produced,^44^ with typical size of 0.5–1 µm.

The presence of magnetite can be explained by its sensitivity to the concentration of Fe(ii).^45^ Meanwhile, the presence of Fe(ii) can be explained by the fact that we started with a Fe(iii) solution, which tends to be reduced to Fe(ii) in such conditions. Our results suggest that magnetite was produced directly during Burkin's experiments and that the sample was not only oxidising prior to their XRD analysis. However, it is difficult to draw direct conclusions from a comparison with their work as the H_2_/Fe molar ratio in our experiments was probably lower: they conducted experiments at up to 68 bar of hydrogen, although they did not specify the initial free volume permitted for gaseous loading in their 2 litres autoclave, with a similar iron concentration than in our experiments (they reached 5 g L^−1^ after their experiments with incomplete reactions).^34^

### 3.4. Degradation of the extractant during HP-HT experiments

Table S2 and Fig. S4 provide an overview of the different compounds detected, their retention times and ions, in the cases of long experiments (16 hours) where most degradation can occur, with Mg(OH)_2_ as a base (V5-2) and NH_3_ as a base (V5-3). The results reported demonstrate that the decomposition of VA10 with ammonia (V5-3) occurred, resulting in the formation of degradation product (#1, Table S2) containing an odd number of N atoms which could coherently correspond to the amide of VA10, RC(O)NH_2_, with a replacement of an –OH group by a –NH_2_ group. The ion with *m*/*z* = 172 in sample V5-3 is not VA10; rather, it is one of the two degradation products formed with ammonia. It also known that some ammonia can complex with VA10, which increases the viscosity.^40^ Conversely, in experiments with Mg(OH)_2_ as the base, peaks of VA10 were still clearly visible. According to the area of the peaks at 12.9 min in ESI− mode (not shown here), the VA10 peak is 1.2 times weaker where ammonia was present, demonstrating more VA10 decomposition according to the peak area ratio. However, as no calibration curve has been drawn up to establish the relationship between concentration and peak area, it is not possible to quantify such degradation here.

## 4. Conclusion

This work demonstrates the influence of hydrogen-stripping on iron precipitation in Versatic Acid 10 at 200 °C and 10 bar H_2_, exploring two bases (NH_3_ and Mg(OH)_2_) and three types of seeds (Ni, Fe, and C). The results show that the presence of a base enhances iron precipitation, with both NH_3_ and Mg(OH)_2_ proving effective. Formation of metallic iron was observed only with Ni seeds under a high H_2_/Fe molar excess (≥12), whereas magnetite was obtained when Mg(OH)_2_ was used as a base with Ni seeds, Fe seeds or without seeds at lower H_2_/Fe molar ratios (≤6.1). In contrast, NH_3_ as a base without seeds and at low H_2_/Fe molar ratio (1.4) led to the formation of both magnetite and hematite. Pure iron metal was obtained only in the presence of nickel seeds, which contradicts the initial aim of isolating iron free from other metals. The use of carbon or iron seeds generally resulted in either no precipitation or the formation of iron oxides. Degradation of VA10 was exclusively observed in the presence of ammonia, despite the absence of quantification of such degradation in the present study. Overall, the H_2_/Fe molar ratio is a critical factor for the formation of metallic iron. Although it is difficult to draw direct conclusions from a comparison with Burkin's work,^33^ as their H_2_/Fe molar ratio is probably higher than in our study, this is consistent with their final emphasis on controlling the reduction rate by transporting hydrogen from the gas phase into the solution.

## Author contributions

Clément Laskar: conceptualisation, investigation, validation, formal analysis, methodology, visualisation, writing – original draft preparation; Koen Binnemans: funding acquisition, conceptualisation, methodology, project administration, resources, supervision, writing – review & editing.

## Conflicts of interest

The authors declare that they have no known competing financial interests or personal relationships that could have appeared to influence the work reported in this paper.

## Supplementary Material

RA-016-D6RA00829A-s001

## Data Availability

The data supporting this article have been included as part of the supplementary information (SI). Supplementary information: whole experimental conditions and results (Table S1), additional SEM-EDX results (Fig. S1 and S2) and XRD results (Fig. S3) when iron oxides are precipitating, HPLC results with degradation products (Table S2 and Fig. S4). See DOI: https://doi.org/10.1039/d6ra00829a.
