# Supplementary material for: Recovery of metallic iron from the loaded organic phase after solvent extraction by precipitation–stripping with hydrogen gas
Source: RSC Adv. 2026 Mar 4;16(14):12374–82. doi: 10.1039/d6ra00829a (PMC12958313; doi:10.1039/d6ra00829a)
Supplement: RA-016-D6RA00829A-s001 [file RA-016-D6RA00829A-s001.pdf]

Supplementary Information (SI) for RSC Advances.
This journal is © The Royal Society of Chemistry 2026

Supplementary Information

Recovery of metallic iron from the loaded organic phase after solvent extraction by precipitation-stripping with hydrogen gas

Clément Laskar[1,2*], Koen Binnemans[1]

[1]Department of Chemistry, KU Leuven, Celestijnenlaan 200F, P.O. Box 2404, 3001 Leuven, Belgium
[2]Laboratoire de Génie Chimique, Université de Toulouse, CNRS, INP, UPS, 4 Allée Emile Monso, 31400, Toulouse, France

E.mail: clement.laskar@toulouse-inp.fr

Table S1 Precipitation yields of Fe and the nature of the precipitated particles were determined after the experiments with different extractants (extr.) and experimental conditions. The initial pressure of H_2 loaded at 25 °C was 10 bar for all experiments.

#	Extr.	$C(Fe)_i$ (g L^{-1})	Fe precipitation (%)	Seeds type	Seeds (mg)	Extr. (vol.%)	Base type	Base (g/L)	Base/Fe excess[a]	H_2/Fe excess[a]	Time (h)	Volume (mL)	Stirring (rpm)	T (°C)	P_i (bar)	P_f (bar)	Precipitated particles XRD	Precipitated particles SEM
V1-1	VA10	2.72±0.01	No	Fe-type I	50	10	–	–	–	23	2	15	600	200	14.2	14.9	N.A.	N.A.
D1-1	D2EHPA	5.55±0.01	4.1±0.3	Fe-type I	50	10	–	–	–	11	2	15	600	150	13.6	14.0	N.A.	N.A.
C1-1	Cyanex 272	10.1±0.01	–[b]	Fe-type I	50	10	–	–	–	6	2	15	600	200	14.8	15.0	N.A.	N.A.
V1-2	VA10	2.67±0.01	38.1±0.3	Fe-type I	50	10	$Mg(OH)_2$	6.8	2.4	23	2	15	600	200	15.4	15.2	N.A.	N.A.
D1-2	D2EHPA	5.30±0.03	12.3±0.1	Fe-type I	50	10	$Mg(OH)_2$	6.8	1.2	12	2	15	600	150	13.9	14.0	N.A.	N.A.
C1-2	Cyanex 272	10.50±0.03	14.3±0.1	Fe-type I	50	10	$Mg(OH)_2$	6.8	0.6	6	2	15	600	200	14.7	14.5	N.A.	N.A.
V2-1	VA10	5.04±0.01	4.1±0.1	Ni	50	10	–	–	–	12	2	15	600	200	14.57	14.99	N.C.	Fe metal
V2-2	VA10	5.04±0.01	12.6±0.3	Ni	50	10	$Mg(OH)_2$	6.8	1.3	12	2	15	600	200	14.84	15.12	N.C.	Fe metal
V2-3	VA10	5.04±0.01	62±1	Ni	100	10	$Mg(OH)_2$	6.8	1.3	6.1	2	30	600	200	15.83	15.93	Fe_3O_4	Fe_3O_4
V2-4	VA10	5.04±0.01	–[b]	Ni	50	10	NH_3	8.7[e]	2.8	12	2	15	600	200	15.36	15.67	N.A.	Fe metal
V2-5[c]	VA10	5.02±0.01	9.2±0.1	Ni	50	30	NH_3	8.7[e]	2.8	12	2	15	600	200	18.56	–	N.C.	Fe metal
V3-1	VA10	5.18±0.03	No	C	16	30	–	–	–	7.3	2	25	600	200	13.57	13.75	N.A.	N.A.
V3-2	VA10	5.18±0.03	3.8±0.2	C	16	30	$Mg(OH)_2$	6.8	1.3	7.3	2	25	600	200	14.62	15.54	B.D.L.	Fe_3O_4
V4-1	VA10	12.18±0.02	28.8±0.3	Fe-type I	20	30	$Mg(OH)_2$	10	0.8	1.9	16	40	1000	200	14.29	14.69	Fe_3O_4	Fe_3O_4
V4-2	VA10	12.18±0.02	28.1±0.4	Fe-type II	20	30	$Mg(OH)_2$	10	0.8	1.9	16	40	1000	200	14.27	14.66	Fe_3O_4	Fe_3O_4
V4-3	VA10	12.18±0.02	30.4±0.4	–	–	30	$Mg(OH)_2$	10	0.8	1.9	16	40	1000	200	13.96	13.31	Fe_3O_4	Fe_3O_4
V5-1[d]	VA10	16.06±0.02	No	–	–	30	–	–	–	1.4	16	40	1000	200	14.10	13.02	N.A.	N.A.
V5-2[d]	VA10	16.06±0.02	21.9±0.1	–	–	30	$Mg(OH)_2$	10	0.6	1.4	16	40	1000	200	14.71	14.88	Fe_3O_4	Fe_3O_4
V5-3[d]	VA10	16.06±0.02	43.4±0.4	–	–	30	NH_3	7.1[e]	0.7	1.4	16	40	1000	200	18.97	15.19	$Fe_2O_3+Fe_3O_4$	$Fe_2O_3+Fe_3O_4$

N.A. means not analysed; N.C. means not conclusive, as the Fe metal and Ni metal peaks overlap in the diffractogram; B.D.L. means below detection limit, as the amount of solid recovered was too low to enable XRD analysis.

[a]Excess ratios are calculated according to the stoichiometry of reactions (4) and (5).

[b]No titration was possible due to the formation of a third phase. In this case, XRD analysis was not feasible.

[c]Decanol (5% vol.) was added as a phase modifier to avoid the formation of a third phase.

[d]The solid was recovered in a glovebox to avoid its oxidation.

[e]The concentration of NH_3 in the solution is calculated by using the ideal gas law, assuming that all the injected NH_3 dissolves in the solution as NH_3 forms a complex with VA10. The pressure decreases to 1 bar after NH_3 loading.

Table S2 The analysed compounds were examined using HPLC in an organic solution following experiments V5-2 and V5-3. Degradation products (#1 and #2) only appeared in experiments with NH_3 as a base. The corresponding spectra are shown in Figure S4.

Compound	Mass (g/mol)	Retention time (min)	m/z (+)	Ion formula	m/z (–)	Ion formula
**Degradation product #1**	171	11.7	172	$[M+H]^+$	–	
			194	$[M+Na]^+$	–	
			204	$[M+MeOH+H]^+$	–	
			226	$[M+MeOH+Na]^+$	–	
			343	$[2M+H]^+$	–	
			365	$[2M+Na]^+$	–	
**Degradation product #2**	185	12.3	186	$[M+H]^+$	–	
			208	$[M+Na]^+$	–	
			218	$[M+MeOH+H]^+$	–	
			240	$[M+MeOH+Na]^+$	–	
**Versatic Acid 10**	172	12.9	304[a]	–	171	$[M-H]^-$
			307[a]	–	217	$[M+HCOOH-H]^-$
					181[a]	–
					317[a]	–
					385[a]	–
					453[a]	–

Degradation product #1 has the molar mass of the amide of VA10 ($RC(O)NH_2$); M(VA10)=172 g/mol; in positive mode (ESI+), no molecular ions M^+ are formed, only ions of the type $[nM^+X]^+$; in negative mode (ESI–) VA10 ionises best because it is an acid.

[a]The exact ion formula is unknown.

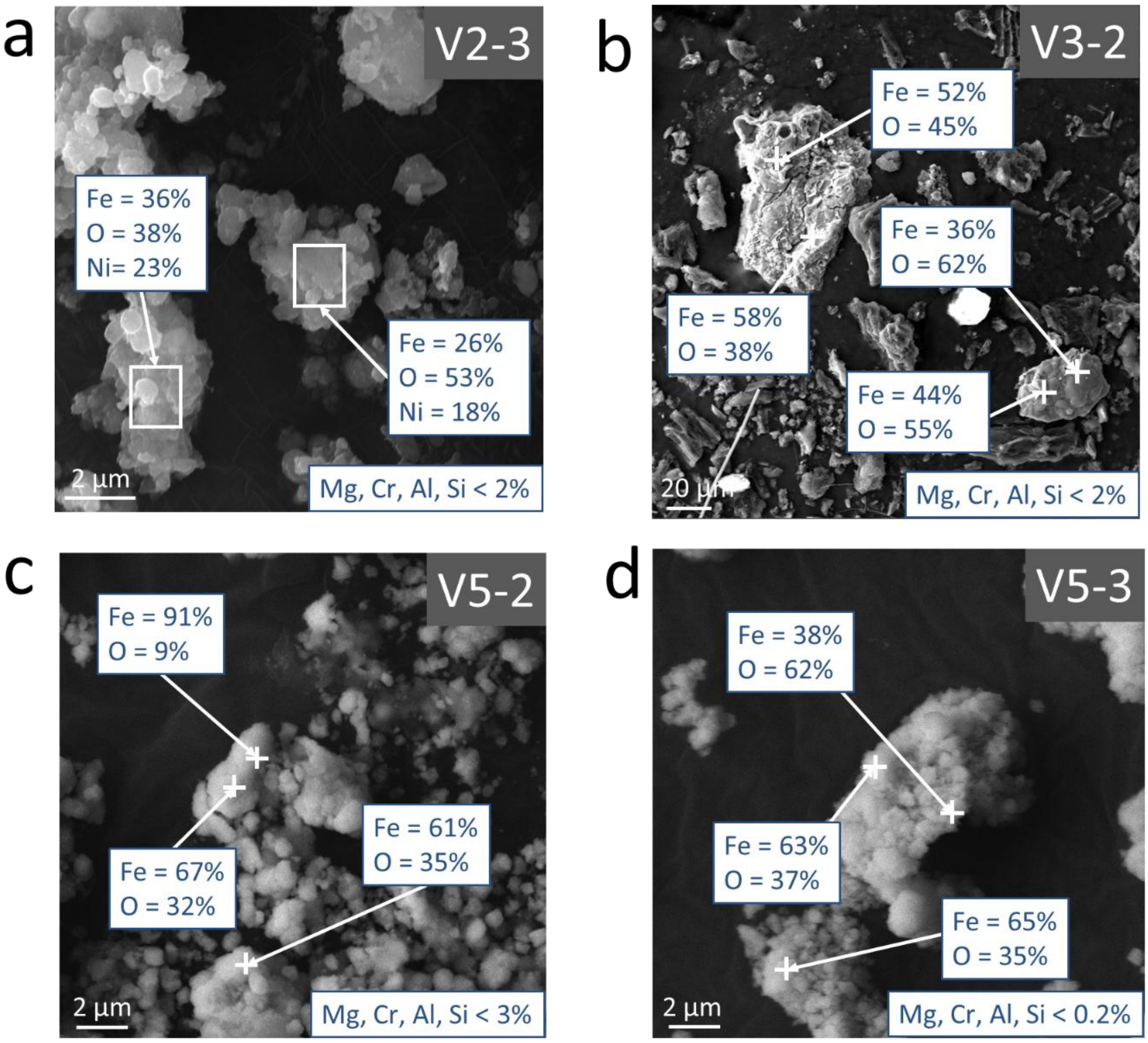

Fig. S1 SEM microphotographs in secondary electron mode (SE mode) with elemental composition analysis performed by EDX. Iron precipitated as iron oxides. See Table S1 for experimental details.

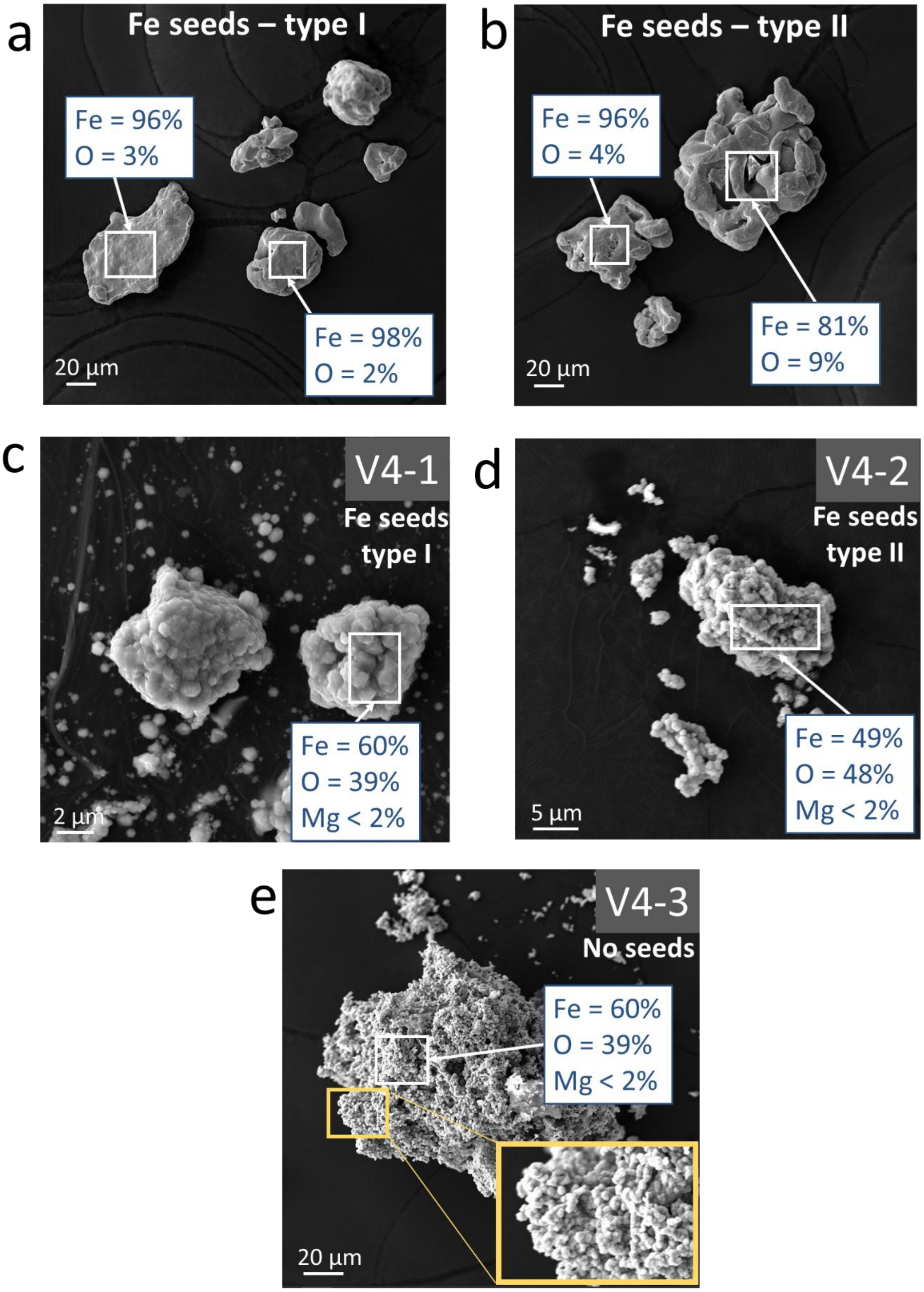

Fig. S2 SEM microphotographs in secondary electron mode (SE mode) of: i) the original Fe seeds **(a,b)** and the precipitate after experiments using these seeds **(c,d)**; ii) precipitate with no seeds added **(e)**. The morphology and particle size distribution of the initial Fe seeds are similar. In all cases, Fe precipitates as iron oxides, with 0.5–1 µm pellets that are characteristic of magnetite material forming larger agglomerates. See Table S1 for experimental details.

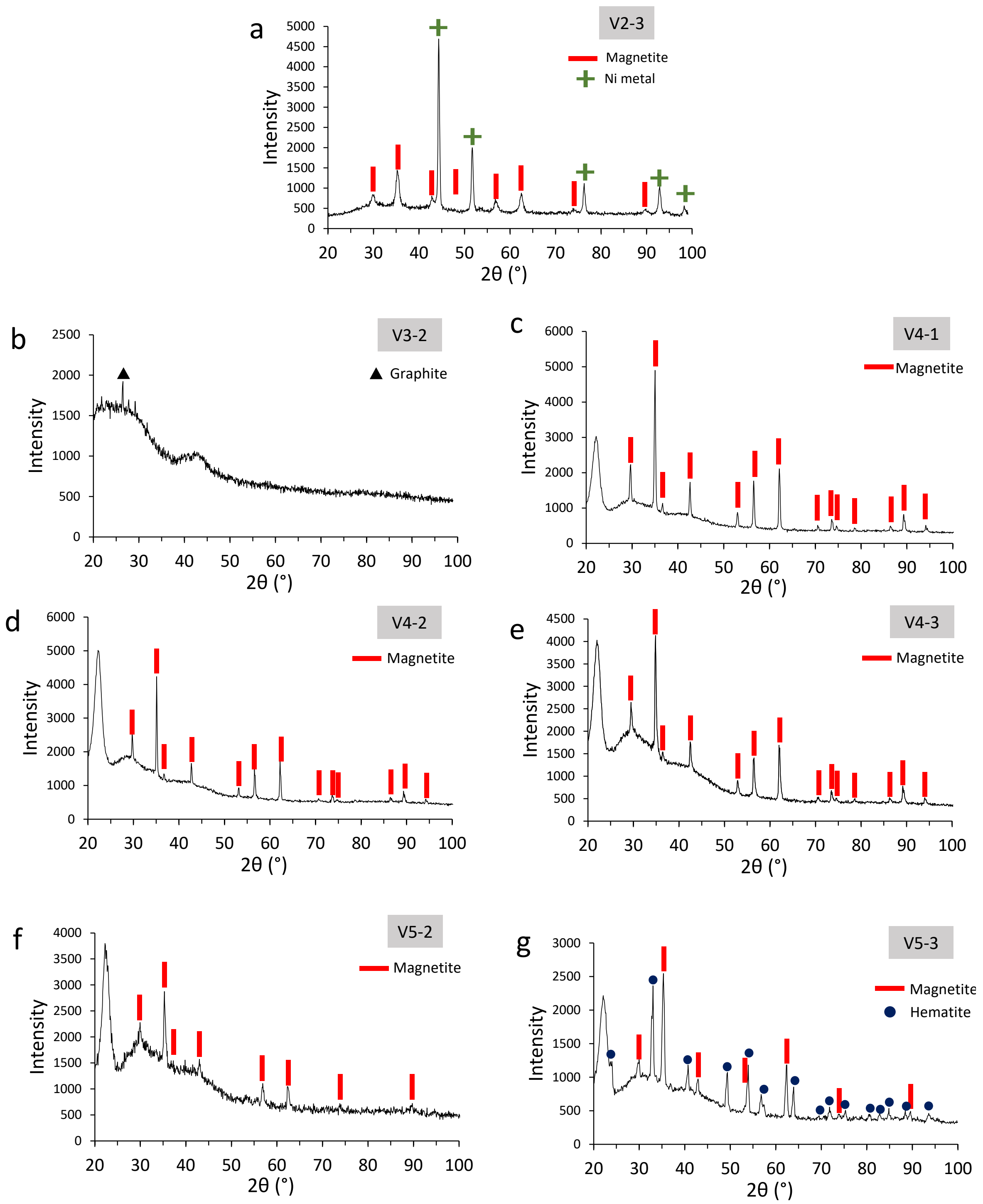

Fig. S3 XRD diffractograms of the precipitate from experiments in which Fe oxides were observed using SEM-EDX. Magnetite and hematite were observed; however, hematite was only present in the presence of NH_3 base. The large peak at 22° is due to the filter through which the solid was analysed in the corresponding experiments. For experiment V3-2, the amount of solid recovered was too low to observe the peaks of the iron oxides; only the main peak of the graphite from the seeds was observed. The structures reported are magnetite (ICSD 082449),[1] hematite (ICSD 082136),[2] and graphite (00-001-0640).[3] See Table S1 for experimental details.

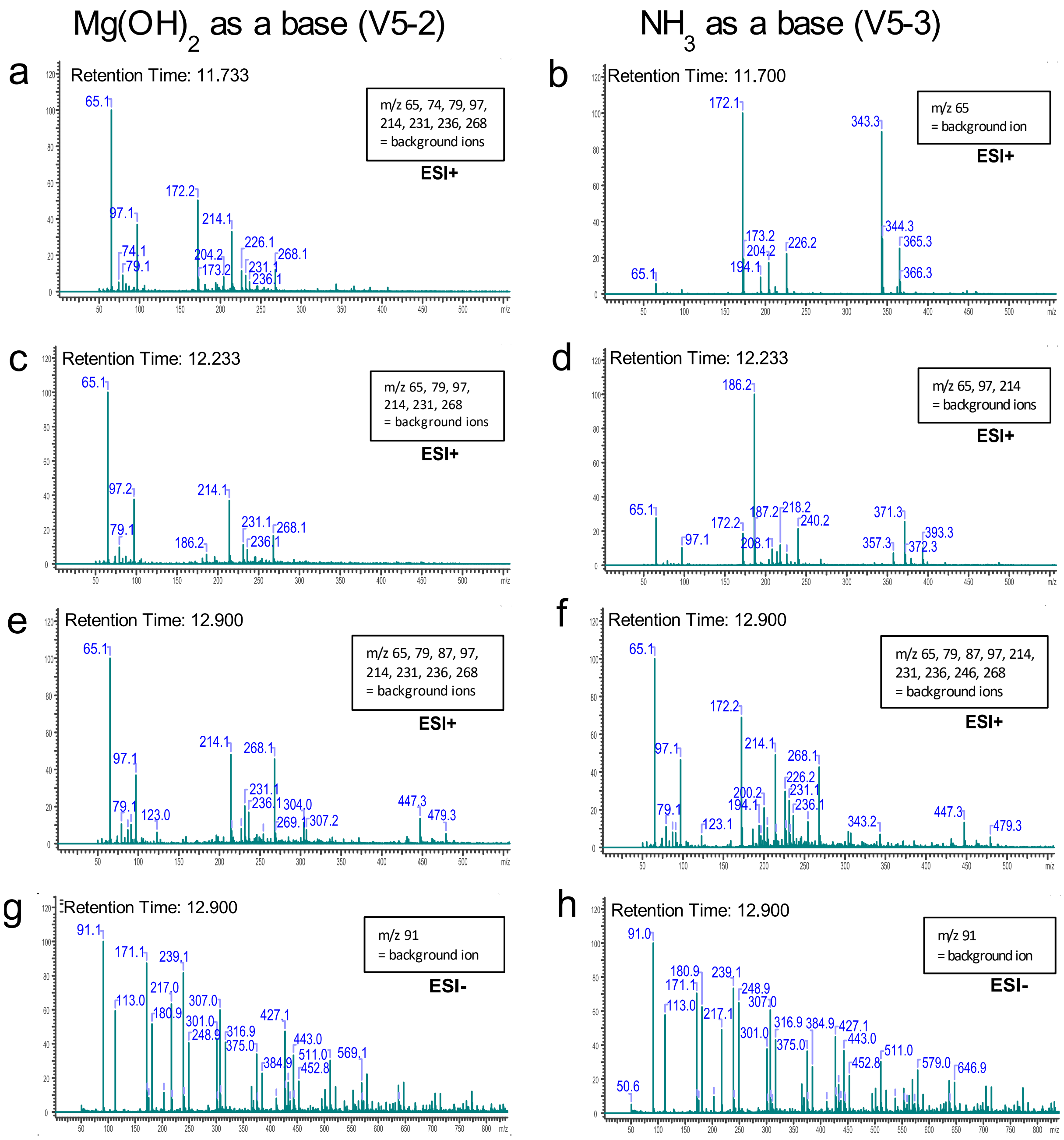

Fig. S4 Comparison between HPLC measurements of organic solution after experiments V5-2 and V5-3, with ESI+ ion mode at retention time of 11.7 min **(a,b)**, 12.2 min **(c,d)** and 12.9 min **(e,f),** and with ESI– ion mode at a retention time of 12.9 min **(g,h)**. The compounds associated with the corresponding peaks are detailed in Table S2.

Supplementary references

1 H. Okudera, K. Kihara and T. Matsumoto, *Structural Science*, 1996, **52**, 450–457.

2 V. A. Sadykov, L. A. Isupova, S. V. Tsybulya, S. V. Cherepanova, G. S. Litvak, E. B. Burgina, G. N. Kustova, V. N. Kolomiichuk, V. P. Ivanov and E. A. Paukshtis, *Journal of Solid State Chemistry*, 1996, **123**, 191–202.

3 J. D. Hanawalt, H. W. Rinn and L. K. Frevel, *Ind. Eng. Chem. Anal. Ed.*, 1938, **10**, 457–512.
